# Associations between non-alcoholic fatty liver disease and cognitive impairment and the effect modification of inflammation

**DOI:** 10.1038/s41598-022-16788-x

**Published:** 2022-07-23

**Authors:** Sunghyuk Kang, Eosu Kim, Hanna Cho, Dae Jung Kim, Hyeon Chang Kim, Sun Jae Jung

**Affiliations:** 1 Department of Preventive Medicine, Yonsei University College of Medicine, Yonsei-ro 50-1, Seodaemun-gu, Seoul, 03722 South Korea; 2 Department of Psychiatry and Institute of Behavioural Science in Medicine, Yonsei University College of Medicine, Seoul, South Korea; 3 Department of Neurology, Gangnam Severance Hospital, Yonsei University College of Medicine, Seoul, South Korea; 4 Department of Endocrinology and Metabolism, Ajou University School of Medicine, Suwon, South Korea; 5 Department of Public Health, Graduate School, Yonsei University, Seoul, South Korea

**Keywords:** Liver diseases, Epidemiology, Dementia, Chronic inflammation

## Abstract

This study aimed to evaluate the association between non-alcoholic fatty liver disease (NAFLD) and cognitive impairment and explore the effect modification by the inflammatory status. A total of 4400 community-based participants aged 50–64 years from the Cardiovascular and Metabolic Disease Etiology Research Center were included in this cross-sectional study. NAFLD was identified as the Fatty Liver Index 30 or higher in the absence of excessive alcohol consumption. Cognitive impairment was defined as the total score of the Mini-Mental State Examination (cutoff 24). The inflammatory status was evaluated using white blood cell (WBC) and high-sensitivity C-reactive protein (hsCRP). Multivariate logistic regression analyses were performed. Stratified analyses by the WBC count (the highest quartile) and the hsCRP level (≥ 1.0 mg/dL vs. < 1.0 mg/dL) were conducted. Participants with NAFLD showed an increased prevalence of cognitive impairment (odds ratio [OR] = 1.26; 95% confidence interval [CI] = 1.04–1.52) compared with the non-NAFLD population. In women, this association was significantly stronger in the highest quartile WBC group than in lower WBC group (OR = 1.81; 95% CI = 1.19–2.74 vs. OR = 1.02; 95% CI = 0.78–1.33, p-interaction = 0.05). NAFLD was positively associated with a higher proportion of cognitive impairment, and this association was stronger in women with higher inflammatory status.

## Introduction

Dementia is a burdensome disease that affects 43.8 million people worldwide^1^ and globally costs 1 trillion US dollars annually^2^. As dementia has a deteriorative and irreversible course, preventive interventions are indispensable to lower this burden. It has been suggested that modifying potential risk factors for dementia, such as less education, hypertension, hearing impairment, smoking, obesity, depression, physical inactivity, diabetes, low social contact, excessive alcohol consumption, traumatic brain injury, and air pollution, might prevent or delay 40% of the dementias^3^. Besides diabetes^4,5^ and obesity^6,7^, metabolic risk factors including hyperlipidemia^8,9^ are known to affect cognitive impairment and play a role in the development of dementias. Metabolic diseases not only cause vascular dementia through cerebrovascular disease but also affects Alzheimer’s disease through insulin resistance^10^ and neuroinflammation^11^.

Non-alcoholic fatty liver disease (NAFLD) is a common metabolic disease, with a global prevalence of 25% in a meta-analysis of studies of populations aged ≥ 18 years in 22 countries^12^. NAFLD is known to be associated not only with liver cirrhosis and cardiovascular disease^13^, but also with psychiatric complications, such as depression, anxiety, and cognitive impairment^14^. NAFLD can be prevented or treated through lifestyle modifications, such as exercise or dietary changes^15^. With this high rate and modifiability of NAFLD, an association with cognitive impairment can have substantial public health implications.

Recently, several studies^16–19^ have investigated the association between NAFLD and impaired cognitive function in the general population, but positive results and negative results coexist. In the United States, a cross-sectional study of 4472 participants in the National Health and Nutritional Examination Survey (NHANES)^16^ showed that NAFLD was associated with lower cognitive performance on the Serial Digit Learning Test. However, in a cross-sectional study of the Framingham Study^19^, participants with NAFLD did not show lower performance on multiple cognitive function tests. To clarify this inconsistency, studies examining the association between NAFLD and cognitive function in a sufficiently large population would be needed. Moreover, to our knowledge, no studies have evaluated the association between NAFLD and cognitive function in the Asian general population. The prevalence of NAFLD in Asia is around 25%^12^, as high as in Western countries. Regarding the emerging importance of NAFLD in Asia, it would be beneficial to confirm the association with cognitive function in a representative Asian general population.

Meanwhile, the biological mechanism underlying the association between NAFLD and cognitive impairment is not yet elucidated. Inflammation is a pivotal mechanism in neuronal injury, the progression of Alzheimer’s disease^11^, and the development of NAFLD and its progression to non-alcoholic steatohepatitis^20^. Inflammation can also affect endothelial dysfunction, insulin resistance, and reactive oxidative stress, which are suggested as possible mechanisms linking NAFLD and cognitive impairment^21^. However, human studies investigating the role of inflammation in relation to the association between NAFLD and cognitive impairment are insufficient.

Therefore, we aimed to evaluate the association between NAFLD and cognitive impairment in a community-based population and investigate the effect modification of the inflammatory status in relation to the association between NAFLD and cognitive impairment.

## Results

Of the 4400 participants included in this study, 1415 (32.2%) of them had Fatty Liver Index (FLI) ≥ 30, defined as NAFLD. 666 (15.1%) participants had Mini-Mental Status Examination (MMSE) < 24, defined as the cognitive impairment. Baseline characteristics according to the NAFLD are presented in Table 1. Participants with NAFLD were older, lesser female, less educated, more current smokers, and had higher proportions of a history of hypertension and diabetes. Participants with NAFLD were more likely to show cognitive impairment (NAFLD group: 233 [17.2%] vs. normal group: 422 [14.1%]; *p* < 0.001). In addition, participants with NAFLD had a higher level of white blood cell (WBC) and high-sensitivity C-reactive protein (hsCRP).

**Table 1:** Descriptive characteristics of the study population from Cardiovascular and Metabolic Diseases Etiology Research Center cohort (N = 4400).

Variables	Total (N = 4400)	Normal: FLI^†^ < 30 (N = 2985, 67.8%)	NAFLD: FLI^†^ ≥ 30 (N = 1415, 32.2%)	*p* value
Age, years, Mean (SD)	57.0 (3.9)	56.8 (3.9)	57.3 (3.8)	< 0.001
Female sex, N (%)	3310 (75.2)	2442 (81.8)	868 (61.3)	< 0.001
**Education, years, N (%)**				0.039
≤ 9	971 (22.1)	629 (21.1)	342 (24.2)	
9–12	2046 (46.5)	1420 (47.6)	626 (44.2)	
12+	1383 (31.4)	936 (31.4)	447 (31.6)	
**Household income, million KRW/year, N (%)**				0.051
≤ 36 (Q1)	1096 (25.0)	712 (24.0)	384 (27.3)	
> 36, ≤ 60 (Q2)	1095 (25.0)	772 (26.0)	323 (22.9)	
> 60, ≤ 84 (Q3)	1095 (25.0)	746 (25.1)	349 (24.8)	
> 84 (Q4)	1095 (25.0)	742 (25.0)	353 (25.1)	
Currently married, N (%)	3845 (87.4)	2598 (87.0)	1247 (88.1)	0.332
Current drinker, N (%)	2649 (60.2)	1778 (59.6)	871 (61.6)	0.220
Current smoker, N (%)	237 (5.4)	110 (3.7)	127 (9.0)	< 0.001
Diabetes mellitus^‡^, N (%)	497 (11.3)	203 (6.8)	294 (20.8)	< 0.001
Hypertension^§^, N (%)	1287 (29.2)	677 (22.7)	610 (43.1)	0.003
Cognitive impairment^¶^, N (%)	666 (15.1)	422 (14.1)	244 (17.2)	0.008
MMSE, Mean (SD)	26.3 (2.6)	26.4 (2.6)	26.1 (2.7)	0.003
WBC, /uL, Mean (SD)	5525 (1502)	5271 (1407)	6060 (1555)	< 0.001
hsCRP, mg/dL, Mean (SD)	1.3 (3.1)	1.1 (2.9)	1.9 (3.4)	< 0.001

^†^FLI = $$100 \times \frac{{e^{{0.953 \times log_{e} \left( {triglycerides} \right) + 0.139 \times \left( {BMI} \right) + 0.718 \times log_{e} \left( {{\upgamma } - {\text{GTP}}} \right) + 0.053 \times \left( {\text{waist circumference}} \right) - 15.745}} }}{{1 + e^{{0.953 \times log_{e} \left( {triglycerides} \right) + 0.139 \times \left( {BMI} \right) + 0.718 \times log_{e} \left( {{\upgamma } - {\text{GTP}}} \right) + 0.053 \times \left( {\text{waist 
circumference}} \right) - 15.745}} }}$$.

^‡^Diabetes was defined as a participant satisfying one of the following three conditions: (1) fasting plasma glucose ≥ 126 mg/dL, (2) glycosylated hemoglobin (HbA1c) ≥ 6.5%, (3) self-reported diabetes diagnosis or the use of anti-diabetic medications.

^§^Hypertension was defined as participants who satisfied one of the following three conditions: (1) systolic blood pressure ≥ 140 mmHg, (2) diastolic blood pressure ≥ 90 mmHg, (3) self-reported diagnosis of hypertension or the use of anti-hypertensive medications.

^¶^Cognitive impairment was defined as MMSE < 24.

NAFLD, non-alcoholic fatty liver disease; FLI, fatty liver index; SD, standard deviation; KRW, Korean Republic won; MMSE, Mini-Mental State Examination; WBC, white blood cell; hsCRP, high-sensitivity C-reactive protein; BMI, body mass index; γ-GTP, gamma-glutamyl transferase.

NAFLD was still significantly associated with cognitive impairment (odds ratio [OR] = 1.26; 95% confidence interval [CI] = 1.04–1.52) after fully adjusting for socio-demographics, lifestyle factors, and comorbidities (Table 2). For the stratification by sex, the association between NAFLD and cognitive impairment was slightly larger in males (OR = 1.39; 95% CI = 0.94–2.07) than in females (OR = 1.21; 95% CI = 0.98–1.51). With stepwise additions of covariates, the ORs for cognitive impairment were altered after adjusting education level, household income, and marital status (OR = 1.35; 95% CI = 1.13–1.62 in model 1 vs. OR = 1.27; 95% CI = 1.05–1.52 in model 2), and this pattern was replicated in sub-groups by sex. The positive association between NAFLD and cognitive impairment was also maintained in the sensitivity analyses, which tested the association between NAFLD and the continuous MMSE score (Supplementary Table 2), the association between the continuous FLI value and cognitive impairment (Supplementary Table 3), and the association between the continuous values of FLI and the MMSE score (Supplementary Table 4).

**Table 2:** Association between non-alcoholic fatty liver disease and cognitive impairment (MMSE < 24).

NAFLD^†^	No. of people	No. (%) of cognitive impairment (MMSE < 24)	OR (95% CI) for cognitive impairment			
			Model 1	Model 2	Model 3	Model 4
**Total (N = 4400)**						
Without NAFLD	2985	422 (14.1)	1.00	1.00	1.00	1.00
With NAFLD	1415	244 (17.2)	**1.35 (1.13–1.62)**	**1.27 (1.05–1.52)**	**1.26 (1.05–1.51)**	**1.26 (1.04–1.52)**
**In men (N = 1090)**						
Without NAFLD	543	59 (10.9)	1.00	1.00	1.00	1.00
With NAFLD	547	73 (13.3)	1.27 (0.88–1.83)	1.36 (0.93–2.00)	1.35 (0.92–1.98)	1.39 (0.94–2.07)
**In women (N = 3310)**						
Without NAFLD	2442	363 (14.9)	1.00	1.00	1.00	1.00
With NAFLD	868	171 (19.7)	**1.38 (1.12–1.68)**	1.23 (0.99–1.52)	1.23 (0.99–1.52)	1.21 (0.97–1.51)

Model 1: Sex, age.

Model 2: Model 1 + education, household income, and marital status.

Model 3: Model 2 + current drinker and current smoker.

Model 4: Model 3 + diabetes and hypertension.

^†^NAFLD was defined as FLI ≥ 30 (FLI = $100 \times \frac{{e^{{0.953 \times log_{e} \left( {triglycerides} \right) + 0.139 \times \left( {BMI} \right) + 0.718 \times log_{e} \left( {{\upgamma } - {\text{GTP}}} \right) + 0.053 \times \left( {\text{waist circumference}} \right) - 15.745}} }}{{1 + e^{{0.953 \times log_{e} \left( {triglycerides} \right) + 0.139 \times \left( {BMI} \right) + 0.718 \times log_{e} \left( {{\upgamma } - {\text{GTP}}} \right) + 0.053 \times \left( {\text{waist circumference}} \right) - 15.745}} }}$).

NAFLD, non-alcoholic fatty liver disease; MMSE, Mini-Mental State Examination; OR, odds ratio; CI, confidence interval; FLI, fatty liver index; BMI, body mass index; γ-GTP, gamma-glutamyl transferase.

Significant values are in bold.

**Figure 1:**
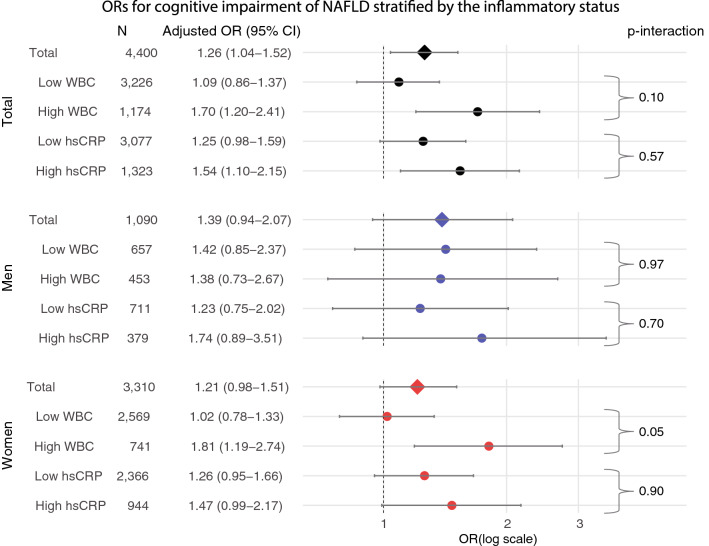
Association between NAFLD and cognitive impairment stratified by the inflammatory status. NAFLD was defined as FLI ≥ 30, and cognitive impairment was defined as MMSE < 24. Each model was adjusted for sex, age, household income, marital status, current drinking status, current smoking status, diabetes, and hypertension. Low WBC: WBC < 75th %tile (6300/μL), High WBC: WBC ≥ 75th %tile (6300/μL). Low hsCRP: hsCRP < 1·0 mg/L, high hsCRP: hsCRP ≥ 1·0 mg/L. NAFLD, non-alcoholic fatty liver disease; WBC, white blood cell; hsCRP, high-sensitivity C-reactive protein; OR, odds ratio; CI, confidence interval; FLI, fatty liver index; MMSE, Mini-Mental State Examination.

In the stratified analyses with inflammatory status, the association between NAFLD and cognitive impairment was significant in participants with a high inflammatory status (high WBC: OR = 1.70; 95% CI = 1.20–2.41, high hsCRP: OR = 1.54; 95% CI = 1.10–2.15), but not in those with a low inflammatory status (low WBC: OR = 1.09; 95% CI = 0.86–1.37, low hsCRP: OR = 1.25; 95% CI = 0.98–1.59) (Fig. 1). The interaction on the association of the WBC strata was statistically significant in women (high WBC: OR = 1.81; 95% CI = 1.19–2.74 vs. low WBC: OR = 1.02; 95% CI = 0.78–1.33, interaction-p = 0.05), but almost absent in men (high WBC: OR = 1.38, 95% CI = 0.73–2.67 vs. low WBC: OR = 1.42, 95% CI = 0.85–2.37, interaction-p = 0.97). The associations in high hsCRP strata were seemed to be stronger than in low hsCRP strata in both men (high hsCRP: OR = 1.74; 95% CI = 0.89–3.51 vs. low hsCRP: OR = 1.23; 95% CI = 0.75–2.02, interaction-p = 0.70) and women (high hsCRP: OR = 1.47; 95% CI = 0.99–2.17 vs. low hsCRP: OR = 1.26; 95% CI = 0.95–1.66, interaction-p = 0.90), but there were no statistical effect modifications.

## Discussion

In this cross-sectional study of the general population aged 50–64 years, participants with NAFLD showed a significant association with cognitive impairment independent of various potential confounders, including socio-demographic factors, lifestyle factors, and comorbidities. The association between NAFLD and cognitive impairment remained significant only in participants with high inflammatory status, not in those with a low inflammatory status.

Using MMSE, this study confirmed the association between NAFLD and cognitive impairment that has been examined in previous studies. In a case–control study conducted in Turkey^17^, the Montreal Cognitive Assessment(MoCA), a screening test for the overall cognitive function, was used to examine the association between NAFLD and cognitive impairment. In this study, 70 hospital-based patients with NAFLD showed 2.99 times higher odds of overall cognitive impairment compared with 73 controls, which was higher than the OR of 1.26 in our study. It is possible that the size of the association in this study was inflated because the control group in this study included hospital staff as well as patients from outpatient clinics. Meanwhile, this difference suggested that NAFLD is more strongly associated with specific cognitive domains. Executive functioning is especially associated with metabolic diseases, such as obesity^22^ and diabetes^23^. Since the MoCA in this study has more emphasis on tasks of frontal functioning, such as executive function and visuospatial function, than the MMSE in our study, there is the possibility that the MoCA was more sensitive to detect cognitive impairment due to NAFLD than the MMSE. Indeed, in this study, NAFLD was only associated with low performance of visuospatial and executive function domains among subdomains of the MoCA. A cross-sectional study^16^ of 4472 adults in the NHANES showed that NAFLD was associated with low performance in the Serial Digit Learning Test but not the other two tests for other cognitive domains. In addition, in a cross-sectional study of 1287 participants in the Framingham Study^19^, there was no association between the presence of NAFLD and cognitive function, but the advanced fibrosis in patients with NAFLD was significantly related to executive function and abstract reasoning among various cognitive function tests. In a cross-sectional study of 1102 participants in the NHANES^18^, participants with both NAFLD and diabetes were associated with lower performance only on Digit Symbol Substitution Test, which evaluates processing speed, sustained attention, and working memory, compared to healthy controls. Taken together, there is the possibility that the cognitive impairment in NAFLD observed in our study might be focused on specific cognitive domains, particularly cognitive functions related to the frontal lobe. Further comprehensive studies on the various cognitive domains affected by NAFLD would be needed.

However, there have been studies^18,19^ demonstrating that NAFLD is not independently associated with cognitive impairment. In these studies, the mean ages of the study population were 69 years^18^ and 61 years^19^, respectively. Compared with our study, in which the maximum age of the study population was 64 years, these negative studies might include a larger proportion of the elderly population. As age increases, the effects of other metabolic and vascular risk factors on dementia tend to weaken^24^. Similarly, the association between NAFLD and cognitive impairment may vary with age. In the study that examined the association between NAFLD and lower performance on the Serial Digit Learning Test^16^, the mean ages of the study population were 37.3 years. Likewise, in the case–control study in Turkey^17^, the mean ages of the case group and the control group were 46.9 years and 43.9 years, respectively. Thus, the association between NAFLD and cognitive function tended to be stronger in the younger population. NAFLD in the middle-aged population may have important implications as a preventive risk factor for dementia.

As intrahepatic fat accumulation progresses, damaged hepatocytes of patients with NAFLD secrete excess pro-inflammatory cytokines^25^. Activated peripheral inflammatory cytokines can activate microglia and increase the permeability of the blood–brain barrier (BBB)^26^, resulting in the transmission of immune cells and neurotoxic factors. These contribute to chronic neuroinflammation and the formation of beta-amyloid plaques^27^, resulting in cognitive impairment through neuronal cell injury or death. The results of our study showed that the association between NAFLD and cognitive impairment remained significant only in the high inflammatory status. This implies the possibility that participants with higher severity of NAFLD were included in the high inflammatory strata due to enhanced systemic inflammation by the liver with steatosis, as well as the possibility that the mechanism of NAFLD causing neuroinflammation was accelerated by the existing inflammation due to other causes. Further studies considering the severity of NAFLD are needed to evaluate the role of inflammation in the association between NAFLD and dementia.

In this study, the effect modification of WBC on the association between NAFLD and cognitive impairment was more pronounced in women. There is no study yet on the sex-related differences in the relationship between NAFLD and cognitive impairment. In women, estrogen deficiency after menopause is suggested as one of the mechanisms of cognitive decline with age^28,29^. Estrogen may have a protective effect on the BBB^30^, suggesting the possibility that the BBB of postmenopausal women is relatively vulnerable to systemic inflammation. In other words, postmenopausal women in the high inflammatory status might be more susceptible to neurotoxic effects of NAFLD transmitted through the BBB. In our study, since most of the women included were postmenopausal, the effect of NAFLD on cognitive impairment in women can be strengthened in the high inflammatory strata. However, further comprehensive studies are needed to investigate the interplay between sex and inflammation.

This study has several strengths. We could utilize a variety of covariate information to build our final model to control for potential confounding factors. In addition, 4400 community participants were analyzed, which is similar to the sample size of the largest of the previous studies^16^. We believe our study utilized the largest sample in the Asian population, evaluating the relationship between NAFLD and cognitive function. With such an adequate sample size, it was possible to demonstrate the association between NAFLD and cognitive impairment in several sensitivity analyses. In addition, this is the first study to investigate the role of inflammation in the association between NAFLD and cognitive impairment.

However, this study has several limitations. First, the definition of NAFLD in this study was based on the FLI, and there was no imaging or histological confirmation. However, as mentioned above, FLI is a widely used surrogate marker of NAFLD and is well validated. Second, the MMSE cannot examine specific domains of cognition which has been associated with NAFLD in a previous study^16^. Further studies using a cognition test battery that comprehensively evaluates multiple cognitive domains would explore the NAFLD-specific cognitive domain. On the other hand, since the MMSE is a screening test that examines the overall cognitive function, it is possible to extend the results of our study to general health care, such as a health examination. Third, since this was a cross-sectional study, it was challenging to guarantee the causality of cognitive impairment due to NAFLD. However, the biological mechanism by which impaired cognitive function can cause NAFLD is rare; therefore, the causality of cognitive impairment caused by NAFLD can be considered. Fourth, participants aged 50–64 years were included in this study; therefore, these results may not be generalizable to the whole geriatric population. Fifth, unmeasured or residual confounding factors may exist, although various potential confounders were adjusted. Sixth, the sample size of the subgroups by sex and inflammatory status was not sufficient to guarantee the statistical power of subgroup analyses. Further studies with a larger sample size would be needed.

## Conclusion

Our results confirmed the association between NAFLD and cognitive impairment independent of potential confounders in a large Asian general population aged 50–64 years. In addition, this association was noticeable with high inflammatory status. Overall, our study highlights the potential promise of NAFLD as an independent risk factor for dementia in the geriatric population, especially in women with high inflammatory status. Further studies with a longitudinal study design in other large populations are needed to replicate our results with causal relationships.

## Methods

### Data source and selection of participants

Data were obtained from the Cardiovascular and Metabolic Diseases Etiology Research Center (CMERC) cohort. The CMERC cohort is a multicenter cohort study aimed at identifying novel risk factors and developing prevention strategies for cardiovascular diseases in Korea^31^. Accordingly, participants who had not been diagnosed with myocardial infarction, stroke, and heart failure during their lifetime were recruited from a community near Seoul, the capital of Korea. From 2013 to 2018, baseline information on the socio-demographic characteristics, medical history, lifestyle factors including alcohol consumption, and psychiatric health of the participants were collected by trained interviewers. In addition, physical examination and blood analysis were conducted on the same day as the interview. Blood samples were collected in the morning after 8 h fasting. A total of 8097 community-based participants aged 30–64 years were recruited at Yonsei University College of Medicine in Seoul, Korea and Ajou University School of Medicine in Suwon, Korea. Among them, 5280 people aged ≥ 50 years underwent the MMSE.

**Figure 2:**
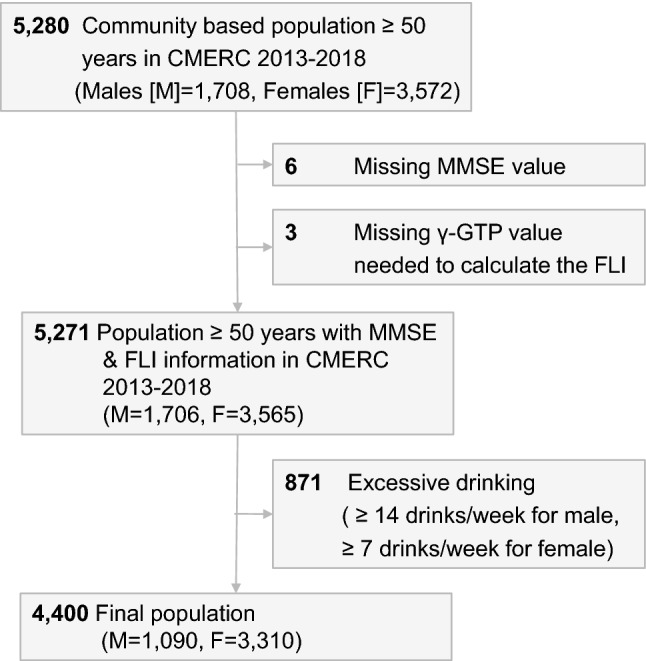
Flowchart of the study population. CMERC, cardiovascular and metabolic diseases etiology research center; MMSE, mini-mental status examination; FLI, fatty liver index; γ-GTP, gamma-glutamyl transferase.

We excluded nine participants with missing information in the MMSE. In addition, gamma-glutamyl transferase (γ-GTP) was used to calculate the Fatty Liver Index (FLI); therefore, three participants without information on γ-GTP were excluded. To rule out alcoholic fatty liver disease, a total of 871 participants with a history of excessive alcohol consumption were excluded. Excessive alcohol consumption was defined as ≥ 14 drinks per week for men or ≥ 7 drinks per week for women^32^ based on self-reports of average alcohol consumption over the past year. The excluded population had different characteristics from the included population by excluding heavy drinkers. (Supplementary Table 1) Included participants were mostly female, older, less educated, and had lower incomes. There were lesser current drinkers and smokers and higher proportions of those with a history of hypertension and diabetes. Finally, 4400 participants aged 50–64 years were included in the analysis (Fig. 2).

### Measurement of the exposure variables and defining NAFLD

NAFLD was defined in this study using the FLI^33^. FLI comprises body mass index (BMI, kg/m^2^), waist circumference (cm), and serum triglyceride (mg/dL) and γ-GTP (IU/L) levels. In this study, height and body weight were measured using stadiometers and a digital weight scale, respectively. Waist circumference was measured using a plastic measuring tape at the midpoint between the lower border of the rib cage and the upper border of the iliac crest during exhalation. Triglycerides and γ-GTP were measured enzymatically using the ADVIA 1800 AutoAnalyzer (Siemens Medical Sol., USA). The formula of the FLI was as follows: FLI = 100 × exp(0.953 × log_e_(triglycerides) + 0.139 × BMI + 0.718 × log_e_(γ-GTP) + 0.053 × waist circumference − 15.745)/[1 + exp(0.953 × log_e_(triglycerides) + 0.139 × BMI + 0.718 × log_e_(γ-GTP) + 0.053 × waist circumference − 15.745)]^33^. According to the formula, FLI can theoretically have a value between 0 and 100. In addition, the application of FLI did not differ by sex.

From the initial utilization of FLI as a criterion for defining NAFLD, FLI has been validated for the diagnosis of NAFLD in a large population, not only in Western countries^34,35^ but also in Asian countries^36,37^. In these validation studies of the Asian population, the area under the receiver operator characteristic curve (AUROC) was higher than 0.8. FLI has been widely used to define NAFLD in the general population of East Asia^38–40^, as is the case of other regions^41–43^. In the European Clinical Practice Guidelines^44^, FLI was suggested as an acceptable alternative for the diagnosis of hepatic steatosis for epidemiologic studies.

Furthermore, we conducted a receiver operator characteristic analysis for FLI on the subgroup of participants to support the validity of FLI. Among 5280 participants aged ≥ 50 years, 2640 people underwent quantitative computed tomography measuring adiposity of liver and spleen. The average value of Hounsfield units (HU) of liver and spleen was measured using a Somatom Definition AS + 128 channel CT (Siemens Healthcare, Forchheim, Germany), a Somatom sensation 64 channel CT (Siemens Healthcare), or a GE Lightspeed VCT apparatus (General Electric Medical System, Milwaukee, WI, USA). With the liver to spleen HU ratio < 1^45^ as the standard diagnosis for fatty liver, the AUROC of FLI was 0.802. (Supplementary Fig. 1).

Although Bedogni et al. suggested FLI ≥ 60 as the cut-off value for NAFLD, validation studies^36,37,46^ in East Asia argued that a lower cut-off value is required, considering the difference in BMI and waist circumference according to ethnicity. The validation study^36^ of the Chinese population aged over 40 years suggested FLI ≥ 30 as an optimal cut-off value; therefore, NAFLD was defined as FLI ≥ 30 in this study.

### Defining cognitive impairment as an outcome variable

MMSE is routinely used as a screening tool in clinical settings to evaluate the global cognitive status^47^. In the CMERC cohort, MMSE was conducted by trained interviewers at baseline. The MMSE comprises 30 items evaluating orientation, attention, memory, language, and visual-spatial functions. Each item was counted as 1 point, and the total MMSE score ranged from 0 to 30. The MMSE was validated in the Korean population, and a cut-off value of 23/24 was suggested for cognitive impairment^48^. Participants with an MMSE score of < 24 were classified as having “cognitive impairment” in this study, based on this cut-off value.

### Measuring the inflammatory status as an effect modifier variable

The WBC count and the hsCRP level were measured and used as a marker of the systemic inflammatory status. The WBC count was determined with flow cytometry using the ADIVIA 2120i (Siemens Medical Sol., USA). Using the serum separated from the collected blood sample, the turbidimetry method using the ADVIA 1800 AutoAnalyzer (Siemens Medical Sol., USA) was used to detect the hsCRP level. We stratified the association between NAFLD and cognitive impairment by the WBC count and the hsCRP level. The WBC count was categorized as high (the highest quartile) and low (other quartiles), and the hsCRP level was categorized as high (≥ 1.0 mg/L) and low (< 1.0 mg/L)^49^.

### Covariates

As mentioned below, structured questionnaires for covariates were queried by trained interviewers in the CMERC baseline survey. Years of education were classified based on the final graduation school: not educated, elementary school, or middle school (education attainment years ≤ 9 years), high school (10–12 years), and university or higher education (> 12 years of education). Participants were also asked about their average annual household income, which was classified into quartiles according to the distribution of the population. Diabetes status was defined as a participant satisfying one of the following three conditions^50^: fasting plasma glucose ≥ 126 mg/dL, glycosylated hemoglobin (HbA1c) ≥ 6.5%, self-reported diabetes diagnosis, or the use of anti-diabetic medications. Plasma glucose and HbA1c levels were measured using the colorimetric method (ADVIA 1800 AutoAnalyzer, Siemens Medical Sol., USA) and high-performance liquid chromatography (Variant II Turbo, Bio-Rad Laboratories, USA), respectively. Hypertension was defined as participants who satisfied one of the following three conditions^51^: systolic blood pressure ≥ 140 mmHg, diastolic blood pressure ≥ 90 mmHg, self-reported diagnosis of hypertension, or the use of antihypertensive medications. Blood pressure was measured in the participant’s right arm using an automated oscillometric device (HEM-7080, Omron Health, Japan) by trained investigators. Systolic and diastolic blood pressures were measured thrice at 2-min intervals, and the mean of the second and third measurements was adopted in this study.

### Statistical analyses

Baseline characteristics by the cognitive impairment status were compared using the t-test and chi-square test. The association between NAFLD and cognitive impairment was evaluated using a multiple logistic regression model. Models were additionally adjusted for (1) age and sex, (2) socio-demographic factors (education level, household income, and marital status), (3) lifestyle factors (current smoking and current drinking), and (4) comorbidities (diabetes and hypertension). A stratification analysis was conducted with cut-off values (highest quartile and 1.0 mg/L, respectively) of the WBC count and the hsCRP level to examine if the association between NAFLD and cognitive impairment differed by the inflammatory status. Since NAFLD and cognitive impairment were defined based on a single cut-off value in the main analyses, sensitivity analyses were performed to consolidate the association between NAFLD and cognitive impairment. The FLI value as a continuous variable was used as an independent variable in the regression analysis. Similarly, a continuous MMSE score was used as a dependent variable in the multiple linear regression. All analyses were conducted using the R software V.4.0.4.^52^.

### Ethics

The protocol of this study was approved by the Institutional Review Board of Yonsei University (YUIRB-4-2013-0661), and written informed consent was obtained from all participants. All procedures in this work complied with the ethical standards of the relevant national and institutional committees on human experimentation and the Helsinki Declaration of 1975.

## Supplementary Information

Supplementary Information.

## Data Availability

The data of the CMERC cohort study can be obtained by submitting an application to the National Biobank of Korea (NBK) and receiving approval from the NBK Access and Sharing Committee. Detailed information is available on the NBK website (http://nih.go.kr/biobank/cmm/main/engMainPage.do). However, we are not permitted to share the data.
