# Supplementary material for: Associations between non-alcoholic fatty liver disease and cognitive impairment and the effect modification of inflammation
Source: Sci Rep. 2022 Jul 23;12:12614. doi: 10.1038/s41598-022-16788-x (PMC9308768; doi:10.1038/s41598-022-16788-x)
Supplement: Supplementary file 1 — Supplementary Information. [file 41598_2022_16788_MOESM1_ESM.docx]

**Supplementary material**

| **Supplementary Table 1.** Comparison of baseline characteristics between included and excluded population | | | |
| --- | --- | --- | --- |
|  | N (%) or Mean (SD) | | |
|  | Included  (N=4,400) | Excluded  (N=880) | p-value |
| Age, years, Mean (SD) | 57.0(3.9) | 56.2(3.8) | <0.001 |
| Female sex, N (%) | 3,310(75.2) | 262(29.8) | <0.001 |
| Education, years, N (%) |  |  | 0.022 |
| ≤9 | 971(22.1) | 163(18.5) |  |
| 9-12 | 2,046(46.5) | 407(46.2) |  |
| 12+ | 1,383(31.4) | 310(35.2) |  |
| Household income,  million KRW/year, N (%) |  |  | <0.001 |
| ≤40 (Q1) | 1,284(29.3) | 180(20.5) |  |
| >40, ≤60 (Q2) | 1,010(23.1) | 212(24.2) |  |
| >60, ≤84 (Q3) | 1,002(22.9) | 223(25.4) |  |
| >84 (Q4) | 1,085(24.8) | 266(29.9) |  |
| Currently married, N (%) | 3,845(87.4) | 789(89.7) | 0.068 |
| Current drinker, N (%) | 2,649(60.2) | 871(99.0) | <0.001 |
| Current smoker, N (%) | 237(5.4) | 223(25.3) | <0.001 |
| Diabetes mellitus, N (%) | 497(11.3) | 126(14.3) | 0.013 |
| Hypertension, N (%) | 1,287(29.2) | 405(46.0) | <0.001 |
| FLI≥30, N (%) | 1,415(37.2) | 538(61.3)^†^ | <0.001 |
| MMSE<24, N (%) | 666(15.1) | 122(14.0)^‡^ | 0.401 |
| ^†^N=877  ^‡^N=874  SD, Standard deviation; KRW, Korean Republic won; FLI, Fatty Liver Index; MMSE, Mini-Mental Status Examination | | | |

| **Supplementary Table 2**. Sensitivity analysis for association between NAFLD (FLI ≥30, binary variable) and MMSE (continuous variable) | | | | | | |
| --- | --- | --- | --- | --- | --- | --- |
| NAFLD | MMSE (continuous) | | | | | |
|  | Model 1 | | Model 2 | | Model 3 | |
|  | β (SE) | p-value | β (SE) | p-value | β (SE) | p-value |
| Total (N=4,400) |  |  |  |  |  |  |
| FLI ≥30=1,415 (32.2%) | -0.225 (0.083) | **0.006** | -0.219 (0.083) | **0.008** | -0.215 (0.085) | **0.012** |
| In men (N=1,090) |  |  |  |  |  |  |
| FLI ≥30=547 (50.2%) | -0.296 (0.145) | **0.041** | -0.283 (0.145) | 0.052 | -0.290 (0.148) | **0.050** |
| In women (N=3,310) |  |  |  |  |  |  |
| FLI ≥30=868 (26.2%) | -0.186 (0.100) | 0.064 | -0.181 (0.100) | 0.071 | -0.170 (0.104) | 0.102 |
| Model 1: Sex, age, education, household income, and marital status  Model 2: Model 1 + current drinker and current smoker  Model 3: Model 2 + diabetes and hypertension  NAFLD, non-alcoholic fatty liver disease; MMSE, Mini-Mental State Examination; SE, standard error; FLI, fatty liver index; | | | | | | |

| **Supplementary Table 3**. Sensitivity analysis for association between FLI (continuous variable) and cognitive impairment (MMSE<24, binary variable) | | | |
| --- | --- | --- | --- |
| FLI | MMSE < 24 | | |
|  | Model 1 | Model 2 | Model 3 |
|  | OR (95% CI) | OR (95% CI) | OR (95% CI) |
| Total (N=4,400) |  |  |  |
| FLI (0-100) | **1.006 (1.001–1.010)** | **1.006 (1.001–1.010)** | **1.006 (1.001–1.010)** |
| In men (N=1,090) |  |  |  |
| FLI (0-100) | 1.005 (0.996–1.013) | 1.004 (0.996–1.013) | 1.005 (0.997–1.014) |
| In women (N=3,310) |  |  |  |
| FLI (0-100) | **1.006 (1.001–1.011)** | **1.006 (1.001–1.011)** | **1.006 (1.000–1.011)** |
| Model 1: Sex, age, education, household income, and marital status  Model 2: Model 1 + current drinker and current smoker  Model 3: Model 2 + diabetes and hypertension  MMSE, Mini-Mental State Examination; FLI, fatty liver index; OR, odds ratio; CI, confidence interval | | | |

| **Supplementary Table 4**. Sensitivity analysis for association between FLI (continuous variable) and MMSE (continuous variable) | | | | | | |
| --- | --- | --- | --- | --- | --- | --- |
| FLI | MMSE (continuous) | | | | | |
|  | Model 1 | | Model 2 | | Model 3 | |
|  | β (SE)(×10^-2^) | p-value | β (SE)(×10^-2^) | p-value | β (SE)(×10^-2^) | p-value |
| Total (N=4,400) |  |  |  |  |  |  |
| FLI (0-100) | -0.487 (0.191) | **0.011** | -0.473 (0.191) | **0.014** | -0.470 (0.201) | **0.020** |
| In men (N=1,090) |  |  |  |  |  |  |
| FLI (0-100) | -0.444 (0.324) | 0.171 | -0.411 (0.207) | 0.207 | -0.432 (0.337) | 0.201 |
| In women (N=3,310) |  |  |  |  |  |  |
| FLI (0-100) | -0.483 (0.237) | **0.041** | -0.470 (0.237) | **0.047** | -0.452 (0.251) | 0.072 |
| Model 1: Sex, age, education, household income, and marital status  Model 2: Model 1 + current drinker and current smoker  Model 3: Model 2 + diabetes and hypertension  MMSE, Mini-Mental State Examination; SE, standard error; FLI, fatty liver index | | | | | | |


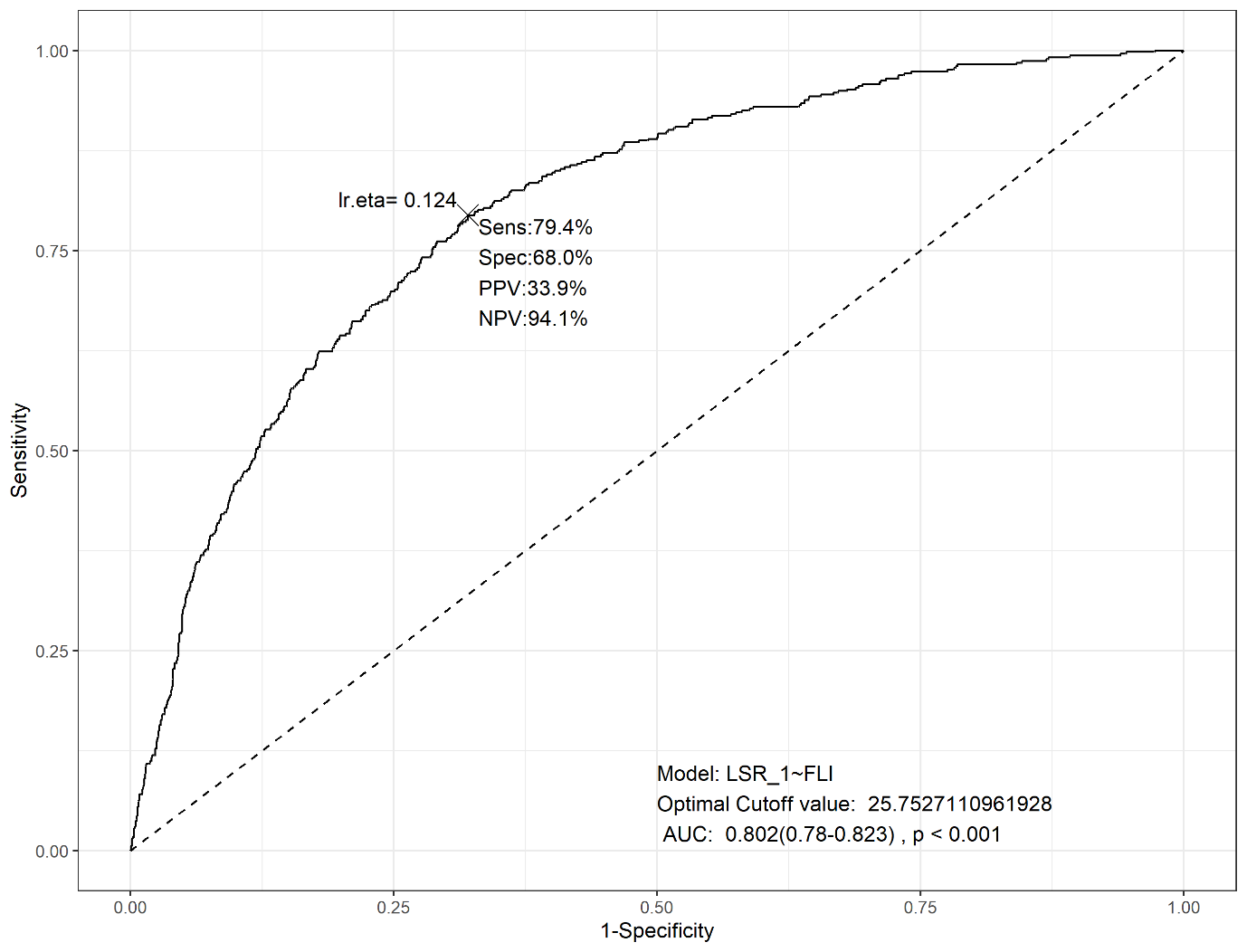


**Supplementary Figure 1**. The receiver operator characteristic curve for fatty liver index in the subgroup of the participants (N=2,640)

The standard diagnosis for fatty liver was defined as the liver to spleen HU ratio < 1.

Fatty liver index ranges from 0 to 100.

Sens, sensitivity; Spec, specificity; PPV, positive predictive value; NPV, negative predictive value; AUC, area under curve;
